# Comparative Analysis of Visual Performance and Optical Quality with a Rotationally Asymmetric Multifocal Intraocular Lens and an Apodized Diffractive Multifocal Intraocular Lens

**DOI:** 10.1155/2020/7923045

**Published:** 2020-04-20

**Authors:** Xiao Wang, Haixia Tu, Yong Wang

**Affiliations:** ^1^Aier School of Ophthalmology, Central South University, Changsha, China; ^2^Aier Eye Hospital of Wuhan University, Wuhan, China

## Abstract

**Purpose:**

To compare the short-term visual outcomes and intraocular optical performance of a rotationally asymmetric multifocal intraocular lens (MIOL) (SBL-3, Lenstec, Inc., Christ Church, Barbados) and an apodized diffractive MIOL (the Acrysof IQ ResTOR SN6AD1, Alcon Laboratories, Inc., Fort Worth, Texas, United States).

**Methods:**

A prospective, comparative, nonrandomized, and single-center study. Sixty-eight age-related cataract patients (81 eyes) after phacoemulsification cataract surgery and in-the-bag MIOL implantation were enrolled. Thirty-eight eyes received SBL-3, and 43 eyes received SN6AD1. Ophthalmological evaluation included uncorrected distance visual acuity (UDVA), uncorrected intermediate visual acuity (UIVA), uncorrected near visual acuity (UNVA), corrected distance visual acuity (CDVA), modulation transfer function (MTF), Strehl ratio (SR), intraocular aberrations (4 mm optical zone), and defocus curve at 3 months postoperatively. The Chinese version of the visual function index-14 (VF-12-CN) and spectacle independence were assessed in all patients.

**Results:**

There was no statistically significant difference between groups in postoperative UDVA (*p* = 0.186). Postoperative UIVA and UNVA were significantly better for the SBL-3 group than for the SN6AD1 group (*p* < 0.01). Statistically significant differences were revealed in defocus levels from –3.50 D to −4.00 D with better visual acuities for the SBL-3 group (*p* < 0.01). For intraocular optical quality outcomes, statistically significant differences between groups were observed in RMS of intraocular total aberrations, coma, and trefoil high-order aberrations, presenting significantly higher values of these parameters in the eyes of the SBL-3 group (p < 0.01). Statistically significant differences were revealed in the MTF values at spatial frequencies of 5 and 10 cycles/degree between groups. There were no significant differences in scores of VF-12-CN, and spectacle independence between the groups (*p* > 0.05).

**Conclusions:**

Both MIOLs were able to successfully restore visual function after cataract surgery. SBL-3 provided better UIVA and UNVA with a wider range of intermediate vision.

## 1. Introduction

Modern cataract surgery with multifocal intraocular lens (MIOL) implantation provides high visual performance and achieves spectacle independence by two or more foci [1]. Many studies have evaluated the efficacy in most cases of MIOL implantation after cataract surgery, including diffractive [2–4], refractive [5–7], or a combination of both methods [8–10]. However, several studies have reported that MIOLs also have limitations, such as unsatisfactory uncorrected intermediate visual acuity (UIVA) [11, 12] and optical side effects, such as glare, halo, and loss of contract sensitivity [13, 14]. To reduce such side effects, refractive rotationally asymmetric MIOL has been introduced into clinical practice. Instead of traditional concentric rings providing different foci, rotationally asymmetric MIOLs have two sectors, a large segment for distance vision, an inferior surface-embedded segment for near vision, and a smooth transition zone between two segments. Fewer transition zones between different power zones should lead to less energy loss and improved contrast sensitivity. Previous reports have indicated that the implantation of rotationally asymmetric MIOLs provided high-quality uncorrected distance and near visual acuities (UDVA and UNVA) and showed high subjective satisfaction and lower spectacle dependence [15, 16]. The first commercially available rotationally asymmetric MIOL, the Lentis Mplus LS-312 (Oculentis GmbH, Berlin, Germany), achieved better UIVA and contrast sensitivity outcomes, whereas the apodized diffractive MIOL (the AcrySof ReSTOR SN6AD3, Alcon Laboratories Inc., Fort Worth, Texas, United States) achieved better UNVA [17]. The SBL-3 MIOL (Lenstec, Inc., Christ Church, Barbados) is another asymmetric MIOL based on the same optical principle of two refractive zones for far and near vision; it has a larger optical area of near segment, without loss of the central aspect, which can be regarded as noncentral-sparing rotationally asymmetrical MIOL. Moreover, as previously reported by McNeely et al., the near visual performance is better with SBL-3 MIOL than with Mplus [18]. However, no studies have compared the visual outcomes and intraocular performance between SBL-3 and apodized diffractive MIOL.

The aim of the study was to compare the visual outcomes and intraocular optical quality of the eyes with the noncentral-sparing refractive rotationally asymmetric SBL-3 MIOL and the eyes with an apodized diffractive MIOL (the Acrysof IQ ResTOR SN6AD1, Alcon Laboratories, Inc., Fort Worth, Texas, United States).

## 2. Patients and Methods

### 2.1. Patients

A total of 68 patients (81 eyes) were enrolled in this prospective, monocentric, nonrandomized, comparative clinical study. The inclusion criteria were patients with age-related cataract, pupil diameter between 2.75 mm and 6.00 mm, an expected postoperative refractive astigmatism of less than 1.0 diopters (D), and absence of any ocular comorbidity that might influence the visual outcome. The exclusion criteria were patients with a history of ocular trauma or intraocular surgery, glaucoma, active ocular diseases, lens dislocation, or corneal disease, such as scars or dystrophy. All patients underwent phacoemulsification followed by MIOL implantation in a capsular bag.

All patients were adequately informed and signed a consent form. The study was adhered to the tenets of the Declaration of Helsinki and approved by the local ethical committee. The patients were informed about the MIOL type implanted and could choose between the two MIOLs. Details about the two MIOLs are provided in Table 1.

### 2.2. Surgical Technique

All surgeries were performed by the same surgeon with standardized phacoemulsification. Continuous curvilinear capsulorhexis with an approximate 5.0 mm diameter was created through a 2.2 mm self-sealing corneoscleral incision. All incisions were placed on the steepest corneal meridian. After the phacoemulsification of the nucleus using the “stop and chop” technique followed by irrigation and aspiration of the cortex (the Centurion Vision System; Alcon Laboratories Inc., Fort Worth, Texas, United States), the foldable MIOL was inserted in the capsular bag using an injector according to the manufacturer's protocol. The SBL-3 MIOL was positioned with near segment placed inferonasally. The remaining viscoelastic was aspired, and the incision was sealed using balanced salt solution. No adverse events occurred.

### 2.3. Preoperative and Postoperative Examinations

The MIOL power was calculated to achieve emmetropia in all eyes. Every patient underwent a detailed preoperative ophthalmologic examination with UDVA and CDVA measurements, Goldmann tonometry, and slit-lamp examination of the anterior and posterior segments. Pentacam (Oculus Inc., Wetzlar, Germany), SS-OCT (DRI-OCT, Topcon, Tokyo, Japan), biometry (LENSTAR LS 900, Haag-Streit AG, Bern, Switzerland), iTrace (Tracey Technologies, Houston, Texas, United States), and OPD Scan-III (NIDEK, Inc., Fremont, California, United States) were used.

Postoperative examinations were performed 3 months after surgery. UDVA and CDVA were measured using logarithm of the minimum angle of resolution (logMAR) charts for distance (5 m) and with Radner reading charts for intermediate and near vision (80 cm and 40 cm). After subjective distance correction, defocus curves were constructed for vergence distances ranging between +1.0 D and −4.0 D in 0.5 D steps. In the defocus curve, 0 D of defocus corresponds to CDVA. Therefore, if minus lenses are added, then the test image is closer to the patient. Therefore, the intermediate vision corresponds with −1.5 D and −1.0 D of defocus. The near vision corresponds with −2.5 D of defocus. All the recorded information was then represented in a 2-dimensional graphic display using Cartesian coordinates (*x*-axis, spherical blur; *y*-axis, distance visual acuity). In addition, the visual function index of life quality and the rate of spectacle independence were evaluated 3 months postoperatively.

Nondilated aberrometry was performed using the OPD Scan-III. Intraocular total aberrations, intraocular high-order aberrations (HOA), coma, trefoil, and spherical aberrations were evaluated under the 4.0 mm optical zone. MTF values at 5, 10, 15, 20, 25, and 30 cpd, as well as SR values were recorded using iTrace with 4.0 mm pupil diameters.

### 2.4. Statistical Analysis

The statistical analysis was performed using only monocular data and the SPSS statistics software package version 23.0 for Windows (SPSS Inc., Chicago, Illinois, United States). The normality of all data samples was evaluated using the Kolmogorov–Smirnov test. The Student's *t*-test was used when the parameters followed a standard normal distribution, whereas the Mann–Whitney test was used to compare the analyzed parameters between groups. For all statistical tests, the same level of significance was used (*p* < 0.05).

## 3. Results

A total of 81 eyes of 68 patients undergoing phacoemulsification were included. According to the type of MIOL implanted, two groups were differentiated: SBL-3 group included 38 eyes implanted with the SBL-3 MIOL and SN6AD1 group included 43 eyes.  Table 2 summarizes the preoperative conditions of the groups of eyes analyzed in the study. As shown, no statistically significant differences were found between age groups (Student's *t*-test; *p* = 0.090). LogMAR UDVA, LogMAR CDVA, sphere, and cylinder were not significantly different in these groups (Mann–Whitney test; *p* ≥ 0.262).

There were no intraoperative and postoperative complications in any case. All the MIOLs were well centered, and there was no case of posterior capsule opacification recorded within the first 3 months. Follow-up was complete in all patients.

### 3.1. Visual and Refractive Outcomes

Table 3 summarizes the visual and refractive outcomes at 3 months after surgery. A significant improvement with surgery in UDVA was observed in both groups (Mann–Whitney test; *p* < 0.01). The mean UDVA was 0.12 ± 0.11 LogMAR in the SBL-3 group and 0.09 ± 0.08 LogMAR in the SN6AD1 group. No significant changes were found postoperatively in the UDVA between groups (Mann–Whitney test; *p* = 0.186). We found statistically significant differences between groups for UIVA and UNVA (Mann–Whitney test; *p* < 0.01). For subjective refraction, significant reductions in the manifest sphere and cylinder were found during the follow-up in both groups (Mann–Whitney test; *p* < 0.01). Significant differences between groups were found after surgery in the manifest sphere (Mann–Whitney test; *p* < 0.01).

**Figure 1:**
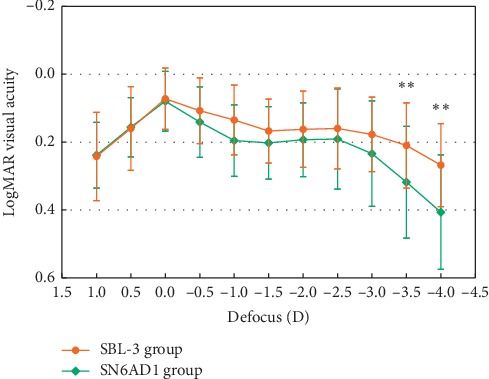
Defocus curve comparison between groups.

Figure 1 shows the mean defocus curve of the patients analyzed in the current study. Both MIOLs provided bimodal profiles showing two peaks of maximum vision at 0.0 D (equivalent to distance vision) and at −2.50 D defocus (equivalent to 40 cm viewing distance from the eye). When the SBL-3 group was compared with the SN6AD1 group, statistically significant differences were revealed in defocus levels from −3.5 D to −4.0 D with better visual acuities for the SBL-3 group (Mann–Whitney tests; *p* < 0.01).

### 3.2. Optical Quality Outcomes

Table 4 shows the internal optical quality outcomes. At 3 months postoperatively, statistically significant differences between groups were observed in RMS of intraocular total aberrations, intraocular high-order aberrations, coma, trefoil, and spherical aberrations; eyes from the SBL-3 group presented significantly higher values of the first four parameters (Student's t and Mann–Whitney tests; p<0.01). In addition, a statistically significant difference in SR was found between both groups (Mann–Whitney test; *p* < 0.01).

**Figure 2:**
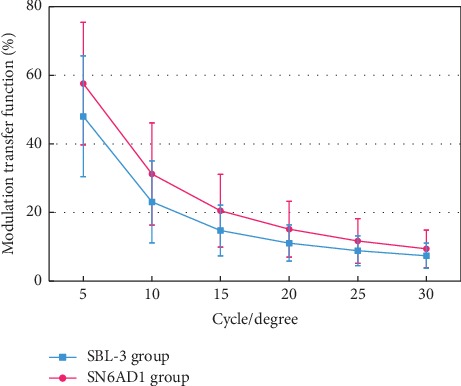
Modulation transfer function (MTF) between groups.

Postoperative MTF curves were obtained for 2 groups. This effect is an average modulation between 0 and 30 cycles/degree (see Figure 2). Statistically significant differences were revealed the MTF values at spatial frequencies of 5 and 10 cycles/degree between groups (Student's *t* and Mann-Whitney tests; *p*<0.05) for a 4 mm pupil diameter (see Table 5).

### 3.3. The QoL Questionnaires of VF-12-CN and the Spectacle Independence

Table 6 shows the Chinese version visual function index-14 (VF-12-CN). Three months postoperatively, the VF-12-CN quality of life questionnaire showed that the average scores of patients with implantation of SBL-3 and SN6AD1 were 1.00 (0.00, 8.00) and 0.80 (0.00, 3.00), respectively. No statistically significant difference between the 2 groups (Mann–Whitney test; *p* = 0.807) was found. There were 3 patients in the SN6AD1 group who had little to moderate difficulty in reading small print or newspaper and a book, while 2 patients in the SBL-3 group had little difficulty in the same situation. Two patients with SBL-3 implantation reported little difficulty in performing handwork, while 1 patient in the SN6AD1 group had a similar complaint. In addition, 1 patient in the SBL-3 group was dissatisfied with reading the nameplate and watching TV. The spectacle independency of the SBL-3 group was 92.85%, and no patients in the SN6AD1 group needed to wear glasses, the spectacle independency was 100%. There was no statistically significant difference between the groups (Student's test; *p* = 0.407).

## 4. Discussion

The restoration of visual function after cataract surgery depends, in part, on the type of MIOL implanted. These MIOLs were designed to provide functional far and near vision. The intermediate vision is acceptable but not as good as the far and near vision [1]. At present, there are 2 types of MIOLs in clinical practice: the traditional rotationally symmetric MIOL and rotationally asymmetric MIOL. The traditional rotationally asymmetric MIOL was characterized by +3.00 D near segment in the anterior optic, which translates to approximately +2.50 D addition at the spectacle plane. And the rotationally symmetric MIOL include 3 different optic designs: diffractive, refractive, and hybrid (combination of refractive and diffractive). The rotationally symmetric MIOLs have already been extensively evaluated for their efficacy in vision restoration. However, visual disturbance may limit the potential benefit with these models [19–21]. Specifically, patients with diffractive MIOL implantation may experience several types of adverse photic phenomena, such as decreased contrast sensitivity, glare, or halos [22]. SBL-3 is the second commercially available model of MIOL based on the concept of rotationally asymmetric. Several studies have shown that this MIOL provided excellent vision outcomes with a good range of functional vision [23].

The present study sought to compare the far, near, and intermediate visual acuity outcomes, defocus curve, and intraocular optical quality parameters in patients with SBL-3 and SN6AD1.

In the current study, a significant improvement in UDVA was observed in both groups, confirming the efficacy of the MIOLs for the visual restoration of the aphakic patient. No statistically significant differences were found in the UDVA between the 2 groups at 3 months postoperatively. In addition, significant differences between groups were found in UIVA and UNVA, with significantly lower LogMAR values in the SBL-3 group. This finding confirmed that SBL-3 provided better UNVA and UIVA than SN6AD1. For manifest refraction, no significant difference between groups was observed in the postoperative manifest cylinder at 3 months after surgery. This result seems logical because 2.2 mm corneoscleral limbal incisions were used in all cases, and the use of microincision cataract surgery provides excellent predictability of postoperative astigmatism [24]. However, a statistically significant difference after surgery in the manifest sphere between groups was found with the better results for the SN6AD1 group. This finding should be considered with caution because of the subjective refraction in patients with rotationally asymmetric MIOL implantation is not completely reliable and difficult to predict because of the optic design with an inferior segmental near addition.

A major objective of the development of MIOL technology is to achieve simultaneous UNVA and UIVA. The defocus curve is a useful procedure to assess the visual performance of a specific model of MIOL using different levels of defocus in 0.50 D steps (equivalent to different viewing distances). In the current study, both MIOLs were able to provide 2 peaks of maximum vision of −2.5 D defocus (equivalent to 40 cm viewing distance from the eye) and at 0.0 D (equivalent to distance vision) with slight drops for intermediate distance. This drop off is much less obvious on the defocus of the SBL-3 group. Moreover, there was a significant difference between both groups in the levels of defocus from −3.5 D to −4.0 D corresponding to the intermediate and near vision with the better results for the SBL-3 group. Interestingly, these findings are consistent with previous studies that found a good range for intermediate vision with SBL-3 [15, 25]. This fact may be attributed to either the smooth optical transition zone between the two areas of the MIOL or some introduction of aberration with this design, providing a larger depth of foci [26].

The RMS of intraocular aberrations, intraocular HOAs, coma, and trefoil aberrations was significantly greater in the SBL-3 group than in the SN6AD1 group in our study. These conclusions are consistent with a previous study in which the implantation of the rotationally asymmetric MIOL increases high-order RMS and primary coma aberration compared with diffractive MIOL postoperatively [15]. This effect seems to be in relation to the rotationally asymmetric design with the gradual transition zone between the two areas. Accordingly, the presence of an intraocular coma and trefoil has been reported in patients implanted with Lentis Mplus, which is usually attributed to its optical geometry [16, 27]. The coma aberration in large values has been considered to have a negative effect on visual acuity because of the visual disturbance it introduces, which may limit the objective optical performance in eyes implanted with rotationally asymmetric MIOL [28]. Interestingly, it is likely that this optical defect with an increase of intraocular aberrations of the rotationally asymmetric allows an extended depth of foci, which contributed to its superiority in UIVA. However, previous studies have found a limited but statistically significant inverse correlation between primary coma and the UNVA [27]. It should be considered that the introduction of coma does not always have a positive effect on the depth of foci. The introduction of the positive amounts of coma or trefoil causes inverse problems and limits vision outcomes in some cases, which were previously due to the imbalance between the blur induced by aberrations and the visual benefit from it [29]. Furthermore, the tilt and decenter of rotationally asymmetric MIOL also caused a larger amount of high-order aberrations. Because of the individual design of rotationally asymmetric MIOL, a central position is crucial to ensure that both near and distance segments of this MIOL provide good visual and optical results [25]. Thus, more research is needed to investigate the correlation between the presence of individual HOAs and visual function.

MTF represents the loss of contrast sensitivity produced by the eye's optics on a sinusoidal grating as a function of its spatial frequency and estimates the performance of the optical systems. Previous studies have shown that eyes with rotationally asymmetric MIOL implantation had significantly better contrast sensitivity results than diffractive rotationally symmetric MIOL [17]. In theory, having fewer transition zones from far vision to near may reduce less light dispersion and improve contrast sensitivity to provide good result of MTF results. However, the introduction of a larger amount of intraocular aberrations may reduce retinal image quality of the eye implanted with SBL-3. This effect may be a design limitation of MIOL design. [30] Nio et al. also found that HOAs may enhance the depth of focus while simultaneously lowering the MTF at higher frequencies. Beyond the direct comparison of MTFs, we also evaluated the SR as a parameter to compare the objective vision quality between the MIOLs provided by iTrace. The SR is a parameter commonly used for estimating the overall optical quality, defined as the ratio of the intensity at the peak of the image formed by an aberrated optical system to the intensity of the aberration-free system [31]. In the current study, the SR parameter indicates that objective vision quality outcomes in the SN6AD1 group were better than those in the rotationally asymmetric MIOL group.

A subjective evaluation of vision quality is necessary to fully understand how individuals perceive their vision. Therefore, this study investigated subjective patient satisfaction through VF-12-CN, which is an applicable tool for evaluating the visual functions of Chinese cataract patients. In our study, the SBL-3 group and the SN6AD1 group had excellent overall QoV scores. There was no statistically significant difference in mean scores between the two groups. A previous study [26] indicated that the overall satisfaction of patients with SBL-3 was high despite some night vision phenomena. However, SBL-3 appeared to provide a better range of visual acuity but had blur vision at distance and near subjects compared to that of SN6AD1 due to the introduction of much undesired intraocular aberrations. At both postoperative assessments, the majority of patients in both groups reported excellent spectacle independency. McNeely et al. [23] found that 100.0% of patients never used spectacles, which is similar to the results in our study. Moreover, a limitation of our study is absence of data on various visual disturbances and photopic phenomena, which should be evaluated in future prospective studies.

## 5. Conclusions

In conclusion, to our knowledge, this study is the first to compare the visual performance, optical performance, and satisfaction of patients between a noncentral-sparing rotationally asymmetrical MIOL and an apodized diffractive MIOL. Both MIOLs provided excellent postoperative outcomes up to 3 months postoperatively. However, eyes with SBL-3 showed better UIVA and UNVA, and eyes with SN6AD1 showed significantly lower intraocular HOAs. Therefore, the rotationally asymmetric MIOL seems to be a promising alternative for MIOL implantation because it provides a wide range of vision acuity and a more physiologic defocus curve. Further studies with rotationally asymmetric design should be performed in the future to enhance or perfect the visual quality to balance the intraocular optical visual defects caused by intraocular HOAs.

## Figures and Tables

**Table 1:** Characteristics of the studied multifocal intraocular lenses (MIOLs).

MIOL	Manufacturer	Material	Optical diameter (mm)	Optics
SBL-3	Lenstec, Inc., Christ Church, Barbados	Acrylic	5.75	Asymmetric refractive, MIOL, biaspheric, +3.0 D
Acrysof IQ ResTOR SN6AD1	Alcon Laboratories, Inc., Fort Worth, Texas, United States	Acrylate/methacrylate copolymer	6.00	Aspheric, apodized diffractive, MIOL, biconvex, +3.0 D

**Table 2:** Preoperative data.

	SBL-3 group mean ± SD (range)	SN6AD1 group mean ± SD (range)	*P* value
Age (years)	58.93 ± 13.30 (26 to 80)	54.24 ± 11.04 (29 to 76)	0.090^*∗*^
LogMAR UDVA	0.79 ± 0.33 (0.30 to 1.40)	0.75 ± 0.38 (0.20 to 1.40)	0.485^*∗∗*^
LogMAR CDVA	0.49 ± 0.23 (0.20 to 1.22)	0.54 ± 0.30 (0.20 to 1.40)	0.689^*∗∗*^
Sphere (D)	−3.40 ± 7.74 (−21.12 to +16.37)	−3.53 ± 6.23 (−22.50 to +3.75)	0.683^*∗∗*^
Cylinder (D)	−0.45 ± 0.18 (−0.75 to −0.25)	−0.49 ± 0.27 (−1.00 to −0.00)	0.262^*∗∗*^
SE	−3.62 ± 7.73 (−21.37 to +16.25)	−3.76 ± 6.25 (−22.88 to +3.50)	0.703^*∗∗*^

^*∗*^Student's *t*-test; ^*∗∗*^Mann–Whitney test. UDVA: uncorrected distance visual acuity; CDVA: corrected distance visual acuity; SE: spherical equivalent; D: diopter; LogMAR: logarithm of the minimum angle of resolution. Corresponding p values for the comparison between groups are shown for each parameter evaluated.

**Table 3:** Postoperative visual and refractive outcomes 3 months after cataract surgery.

	SBL-3 group mean ± SD (range)	SN6AD1 group mean ± SD (range)	*p* value (Mann–Whitney test)
LogMAR UDVA	0.12 ± 0.11 (−0.08 to 0.40)	0.09 ± 0.08 (0.00 to 0.30)	0.186^*∗∗*^
LogMAR UIVA	0.10 ± 0.09 (0.02 to 0.40)	0.43 ± 0.22 (0.10 to 1.00)	<0.001^*∗∗*^
LogMAR UNVA	0.17 ± 0.13 (0.02 to 0.40)	0.34 ± 0.20 (0.10 to 0.80)	<0.001^*∗∗*^
Sphere (D)	−0.33 ± 0.23 (−0.25 to 0.50)	−0.22 ± 0.22 (−0.25 to 0.50)	<0.001^*∗∗*^
Cylinder (D)	−0.29 ± 0.28 (−0.50 to 0.00)	−0.38 ± 0.17 (−0.75 to 0.00)	0.293^*∗∗*^
SE	−0.27 ± 0.28 (−0.75 to +0.25)	−0.09 ± 0.14 (−0.25 to +0.25)	0.030^*∗∗*^

^*∗∗*^Mann–Whitney test. UDVA: uncorrected distance visual acuity; UIVA: uncorrected intermediate visual acuity; UNVA: uncorrected near visual acuity; SE: spherical equivalent; D: diopter; LogMAR: logarithm of the minimum angle of resolution. Corresponding p values for the comparison between groups are shown for each parameter evaluated.

**Table 4:** Postoperative internal aberrations and Strehl ratio (SR) 3 months after cataract surgery.

	SBL-3 group mean ± SD (range)	SN6AD1 group mean ± SD (range)	*p* value
Total aberration	1.28 ± 0.35 (0.78 to 2.03)	0.58 ± 0.29 (0.23 to 1.48)	<0.001^*∗*^
HOAs	0.65 ± 0.08 (0.39 to 0.76)	0.21 ± 0.09 (0.10 to 0.53)	<0.001^*∗∗*^
Coma	0.16 ± 0.05 (0.10 to 0.34)	0.11 ± 0.06 (0.03 to 0.28)	<0.001^*∗*^
Trefoil	0.59 ± 0.11 (0.33 to 0.74)	0.12 ± 0.08 (0.04 to 0.37)	<0.001^*∗∗*^
SA	0.05 ± 0.05 (0.00 to 0.19)	0.06 ± 0.03 (0.17 to 0.33)	0.006^*∗∗*^
SR	0.01 ± 0.01 (0.00 to 0.03)	0.07 ± 0.03 (0.02 to 0.13)	<0.001^*∗∗*^

^*∗*^Student's *t*-test; ^*∗∗*^Mann–Whitney test. HOAs: high-order aberrations; SA: spherical aberration. Corresponding *p* values for the comparison between groups are shown for each parameter evaluated.

**Table 5:** Modulation transfer function 3 months after cataract surgery.

	Modulation transfer function (MTF)		*p* value
	SBL-3 group mean ± SD (range)	SN6AD1 group mean ± SD (range)	
5 cpd	0.048 ± 0.175 (0.027 to 0.732)	0.575 ± 0.178 (0.255 to 0.807)	0.028^*∗*^
10 cpd	0.231 ± 0.119 (0.010 to 0.625)	0.312 ± 0.148 (0.143 to 0.544)	0.032^*∗∗*^
15 cpd	0.147 ± 0.073 (0.007 to 0.409)	0.205 ± 0.106 (0.082 to 0.392)	0.053^*∗∗*^
20 cpd	0.110 ± 0.052 (0.005 to 0.270)	0.151 ± 0.081 (0.058 to 0.309)	0.105^*∗∗*^
25 cpd	0.088 ± 0.043 (0.004 to 0.197)	0.116 ± 0.064 (0.043 to 0.239)	0.210^*∗∗*^
30 cpd	0.074 ± 0.036 (0.004 to 0.168)	0.094 ± 0.054 (0.033 to 0.193)	0.315^*∗∗*^

^*∗*^Student's *t*-test; ^*∗∗*^Mann–Whitney test. cpd: cycle per degree.

**Table 6:** Chinese version of visual function index-14 (VF-12-CN).

Items	No	With a little difficulty	With a moderate amount of difficulty	With a great deal of difficulty	Unable to do the activity
Reading small print, such as labels on medicine bottles, a telephone book, a price tag, bank documents, water, and electricity bill					
Reading a newspaper or a book					
Reading a large-print book or large-print newspaper or numbers on a telephone					
Recognizing people when they are close to you					
Seeing steps, stairs, or curbs					
Reading the nameplate (traffic signs, street signs, or store signs)					
Doing handwork like sewing, knitting, using hand tools?					
Filling out forms or signing names					
Playing games such as mahjong, card games, chess					
Taking part in sports (walking, square dancing, Tai Ji)					
Cooking					
Watching television?					

No difficulty: 0 points; with a little difficulty: 1 point; with a moderate amount of difficulty: 2 points; with a great deal of difficulty: 3 points: unable to do the activity: 4 points.

## Data Availability

The data used to support the findings of this study are available from the corresponding author upon request.

## References

[1] Alio J. L., Plaza-Puche A. B., Férnandez-Buenaga R., Pikkel J., Maldonado M. (2017). Multifocal intraocular lenses: an overview. *Survey of Ophthalmology*.

[2] Packer M., Chu Y. R., Waltz K. L. (2010). Evaluation of the aspheric tecnis multifocal intraocular lens: one-year results from the first cohort of the food and drug administration clinical trial. *American Journal of Ophthalmology*.

[3] Cillino S., Casuccio A., Di Pace F. (2008). One-year outcomes with new-generation multifocal intraocular lenses. *Ophthalmology*.

[4] Alfonso J. F., Fernández-Vega Cueto L., Señaris A., Montés-Micó R. (2007). Quality of vision with the Acri. Twin asymmetric diffractive bifocal intraocular lens system. *Journal of Cataract and Refractive Surgery*.

[5] Chiam P. J. T., Chan J. h., Haider S. I. (2007). Functional vision with bilateral ReZoom and ReSTOR intraocular lenses 6 months after cataract surgery. *Journal of Cataract and Refractive Surgery*.

[6] Pepose J. S., Qazi M. A., Davies J. (2007). Visual performance of patients with bilateral vs combination crystalens, ReZoom, and ReSTOR intraocular lens implants. *American Journal of Ophthalmology*.

[7] Hayashi K., Yoshida M., Hayashi H. (2009). All-distance visual acuity and contrast visual acuity in eyes with a refractive multifocal intraocular lens with minimal added power. *Ophthalmology*.

[8] Alió J. L., Kaymak H., Breyer D., Cochener B., Plaza-Puche A. B. (2018). Quality of life related variables measured for three multifocal diffractive intraocular lenses: a prospective randomised clinical trial. *Clinical & Experimental Ophthalmology*.

[9] Kohnen T., Allen D., Boureau C. (2006). European multicenter study of the AcrySof ReSTOR apodized diffractive intraocular lens. *Ophthalmology*.

[10] Kohnen T., Nuijts R., Levy P., Haefliger E., Alfonso J. F. (2009). Visual function after bilateral implantation of apodized diffractive aspheric multifocal intraocular lenses with a +3.0 D addition. *Journal of Cataract and Refractive Surgery*.

[11] Eckhardt H. B., Hütz W. W., Röhrig B., Grolmus R. (2008). Intermediate vision and reading speed with array, Tecnis, and ReSTOR intraocular lenses. *Journal of Refractive Surgery*.

[12] Blaylock J. F., Si Z., Vickers C. (2006). Visual and refractive status at different focal distances after implantation of the ReSTOR multifocal intraocular lens. *Journal of Cataract and Refractive Surgery*.

[13] Woodward M. A., Randleman B. J., Stulting D. R. (2009). Dissatisfaction after multifocal intraocular lens implantation. *Journal of Cataract and Refractive Surgery*.

[14] Hofmann T., Zuberbuhler B., Cervino A., Montés-Micó R., Haefliger E. (2009). Retinal straylight and complaint scores 18 months after implantation of the AcrySof monofocal and ReSTOR diffractive intraocular lenses. *Journal of Refractive Surgery*.

[15] Jorge L. A., Ana B. P., Jaime J. (2012). Comparison of a new refractive multifocal intraocular lens with an inferior segmental near add and a diffractive multifocal intraocular lens. *Ophthalmology*.

[16] Alió J. L., Piñero D. P., Plaza-Puche A. B., Chan M. J. R. (2011). Visual outcomes and optical performance of a monofocal intraocular lens and a new-generation multifocal intraocular lens. *Journal of Cataract and Refractive Surgery*.

[17] Alió J. L., Plaza-Puche A. B., Javaloy J., José Ayala M. (2012). Comparison of the visual and intraocular optical performance of a refractive multifocal IOL with rotational asymmetry and an apodized diffractive multifocal IOL. *Journal of Refractive Surgery*.

[18] McNeely R. N., Pazo E., Spence A. (2017). Visual quality and performance comparison between 2 refractive rotationally asymmetric multifocal intraocular lenses. *Journal of Cataract and Refractive Surgery*.

[19] de Vries N. E., Webers C. A. B., Touwslager W. R. H. (2011). Dissatisfaction after implantation of multifocal intraocular lenses. *Journal of Cataract and Refractive Surgery*.

[20] Alfonso J. F., Puchades C., Fernández-Vega L. (2009). Visual acuity comparison of 2 models of bifocal aspheric intraocular lenses. *Journal of Cataract and Refractive Surgery*.

[21] Walkow T., Klemen U. M. (2001). Patient satisfaction after implantation of diffractive designed multifocal intraocular lenses in dependence on objective parameters. *Graefe’s Archive for Clinical and Experimental Ophthalmology*.

[22] Montés-Micó R., Alió J. L. (2003). Distance and near contrast sensitivity function after multifocal intraocular lens implantation. *Journal of Cataract and Refractive Surgery*.

[23] McNeely R. N., Pazo E., Spence A. (2017). Visual outcomes and patient satisfaction 3 and 12 months after implantation of a refractive rotationally asymmetric multifocal intraocular lens. *Journal of Cataract and Refractive Surgery*.

[24] Wang L., Xiao X., Zhao L. (2017). Comparison of efficacy between coaxial microincision and standard-incision phacoemulsification in patients with age-related cataracts: a meta-analysis. *BMC Ophthalmology*.

[25] Moore J. E., McNeely R. N., Pazo E. E., Moore T. C. B. (2017). Rotationally asymmetric multifocal intraocular lenses: preoperative considerations and postoperative outcomes. *Current Opinion in Ophthalmology*.

[26] Venter J. A., Barclay D., Pelouskova M., Bull C. E. L. (2014). Initial experience with a new refractive rotationally asymmetric multifocal intraocular lens. *Journal of Refractive Surgery*.

[27] Ramón M. L., Piñero D. P., Pérez-Cambrodí R. J. (2012). Correlation of visual performance with quality of life and intraocular aberrometric profile in patients implanted with rotationally asymmetric multifocal IOLs. *Journal of Refractive Surgery*.

[28] Applegate R., Khemsara V., Sarver E. (2002). Are all aberrations equal?. *Journal of Refractive Surgery*.

[29] Akondi V., Pérez-Merino P., Martinez-Enriquez E. (2017). Evaluation of the true wavefront aberrations in eyes implanted with a rotationally asymmetric multifocal intraocular lens. *Journal of Refractive*.

[30] Nio Y. K., Jansonius N. M., Fidler V., Geraghty E., Norrby S., Kooijman A. C. (2002). Spherical and irregular aberrations are important for the optimal performance of the human eye. *Ophthalmic & Physiological Optics: The Journal of the British College of Ophthalmic Opticians (Optometrists)*.

[31] Di´az-Douto´n F., Benito A., Pujol J. (2006). Comparison of the retinal image quality with a Hartmann-Shack wavefront sensor and a double-pass instrument. *Investigative Ophthalmology & Visual Science*.

